# Clinicopathological Features and Treatment Challenges in Triple Negative Breast Cancer Patients: A Retrospective Cohort Study

**DOI:** 10.5146/tjpath.2020.01516

**Published:** 2021-05-15

**Authors:** Amira Elwan, Aziza E. Abdelrahman, Ahmed A. Alnagar, Mohamed I Abdelhamid, Nashwa Nawar

**Affiliations:** Department of Clinical Oncology and Nuclear Medicine, Zagazig University, Faculty of Medicine, Zagazig, Egypt; Department of Pathology, Zagazig University, Faculty of Medicine, Zagazig, Egypt; Department of Medical Oncology, Zagazig University, Faculty of Medicine, Zagazig, Egypt; Department of General Surgery, Zagazig University, Faculty of Medicine, Zagazig, Egypt

**Keywords:** Triple negative breast cancer, Androgen receptor, Capecitabine, Bicalutamide

## Abstract

***Objective:*** As the genetic and molecular profiles of triple negative breast carcinoma (TNBC) are elucidated, multiple therapeutic targets have been produced. TNBC with less than 1% androgen receptor (AR) expression may respond to enzalutamide with greater response association in higher levels. A metronomic dose of capecitabine and docetaxel are effective developed drugs for angiogenic process inhibition. We aimed to demonstrate the treatment outcome of triple-negative breast cancer patients in correlation to their clinicopathological features.

***Materials and Methods: *** A retrospective cohort study of 80 TNBC patients was conducted. The patients underwent proper observation with the reporting of their treatment and follow-up data. Patients with a metastatic disease, neoadjuvant chemotherapy, follow-up drop or data shortage were excluded from the survival analysis.

***Results: *** The study results revealed a significant association between negative androgen expression and younger age ≤35 years, premenopausal status, higher grade, extracapsular extension, lymphovascular invasion, Ki 67, and CA15-3 (p=0.003, 0.02, <0.001, 0.001, 0.027, 0.005, 0.009 respectively). The three-year overall survival (OS) in patients who received bicalutamide was better than those patients who received capecitabine or docetaxel but of no significance (p=0.46). The three-year disease free survival (DFS) was significantly better in the bicalutamide arm versus the other two groups (p=0.012).

***Conclusions: *** We concluded that extended adjuvant antiandrogen such as bicalutamide and metronomic capecitabine are well tolerated with accepted compliance and affordability compared to docetaxel and are warranted for problem-solving and better DFS and OS in some TNBC patients.

## INTRODUCTION

Triple-negative breast cancer (TNBC) that represents 12-17% of all breast cancers (BC) is defined by less than 1% of the estrogen receptor (ER) and progesterone receptor expression, and normal human epidermal growth factor receptor 2 (HER2) gene copy number and expression (1).TNBCs have more aggressive behavior than non-TNBCs. Patients with TNBC tend to have higher relapse rates and probability of CNS and visceral metastases than those with non-TNBC (2).

Different genomic and molecular technique applications have revealed TNBC heterogeneity in the form of basal-like (BL), immunomodulatory (IM), mesenchymal (M), and luminal androgen receptor (LAR) subtypes, and each one demonstrates a unique pattern of gene expression. Because of the elucidated genetic and molecular profiles of TNBC, multiple therapeutic targets have been produced and TNBCs are amenable for treatment intervention (3).

Anthracycline and taxane-based protocols of chemotherapy were considered as the mainstay treatment of TNBC patients (4). Treatment guidelines of early TNBC patients did not include platinum agents, but their use is explained in specific cases, such as those with a high risk of relapse and in need of rapid disease control, where the use of carboplatin was recommended for patients with known mutant BRCA; however, a carboplatin-based combination is one of the available protocols for adjuvant treatment nowadays (5).

Androgen receptors include 3 domains consisting of amino-terminal domain, DNA binding domain, and a carboxyl-terminal domain that functionally act with each other. The first one is the largest and responsible for the activation of function domain AF1 that includes the tau 1 and tau 2 transcription activating units essential for androgen receptor activity. The amino-terminal domain contains a polyglutamine (CAG) sequence with various repetition numbers (6). Rebbeck et al. have discovered the relationship between patients carrying at least one AR allele with more than 28 CAG repeats and a significant risk of breast cancer (7).

Androgen receptor (AR) is expressed in 12-55% of TNBC cases (8–10). Some variation in expression frequency between studies is due to the different use of anti-AR antibodies or an assay cutoff difference (1% versus 10%). BC with less than 1% AR expression may respond to enzalutamide and may be associated with greater response in higher levels of AR expression (8). In AR-positive TNBC subtype patients, bicalutamide is well tolerated and could be proposed as an alternative to cytotoxic chemotherapy in such patients with better OS and DFS outcomes (11).

In comparison to hormone receptor-positive breast cancer, capecitabine has shown differential activity in TNBC in limited reported data (12). The proposal of metronomic chemotherapy is defined by the close and the regular intervals of chronic administration of low doses of cytotoxic drugs with no prolonged drug-free interruptions, in favor of lower toxicity and risk of drug-resistant tumor cell emergence in comparison to conventional administration (13). TNBC is considered a highly proliferative tumor with more enhanced angiogenesis that supports rapid growth and early metastasis, and tends to have high levels of vascular endothelial growth factor (VEGF). The metronomic dose of capecitabine is effective in TNBC as it leads to inhibition of the angiogenic process (14).

Docetaxel therapy has a significant role in both neoadjuvant and adjuvant management of triple negative breast cancer patients (15). Metronomic administration of docetaxel has achieved survival gains (16).

Compared to non-TNBC cases, TNBC cases are characterized by higher levels of VEGF and the blockade of angiogenesis will therefore lead to improving the outcomes in such patients. This was investigated in adjuvant phase III trials that evaluated the addition of one year of metronomic cyclophosphamide, methotrexate CM maintenance therapy (International Breast Cancer Study Group-22-00), as well as bevacizumab for one year proposed as standard chemotherapy (BEATRICE Study) (17). In this study, we aimed to demonstrate the outcome of triple-negative breast cancer patients treated with various strategies in correlation to their clinicopathological features.

## MATERIAL and METHODS

Eighty TNBC patients were conducted to general surgery, pathology, clinical oncology, and medical oncology departments as a multidisciplinary team in a retrospective cohort study from January 2016 to January 2020. The patient data were collected from the patient’s records with approval by the local ethics committee (Approval no: 6394-15-09-2020, Date: 15.09.2020). Focusing on the patient’s clinical outcome post adjuvant treatment period as extending treatment. Patients with metastatic disease, neoadjuvant chemotherapy proposal, follow-up drop, and data shortage were excluded from the survival analysis. Included patients underwent proper observation with reporting of their treatment and follow-up data, besides the proper history and physical examinations. Full lab, chest x-ray, pelvic abdominal ultrasonography, mammography, breast ultrasound, CT chest, abdomen, and pelvis with contrast, CT brain or MRI with contrast were requested. A bone scan was requested according to the clinical conditions such as bone pain in the early stage and was performed in all local advanced and metastatic cases. Some patients underwent a PET scan. At the general surgery department, the patients underwent either a true-cut or excisional biopsy, breast conservation, or modified radical mastectomy. Eighty patients were proposed adjuvant chemotherapy and 71 patients out of 80 received adjuvant radiotherapy.

### Immunohistochemistry

The staining was carried out using the polymer Envision detection system the Dako EnVision™ kit (Dako, Copenhagen, Denmark). Tissue sections (3–5 µm) were deparaffinized in xylene and rehydrated in graded alcohol. To block endogenous peroxidase, slides were incubated for 10 min in 3% hydrogen peroxide. Dako target antigen retrieval solution (pH 6.0) was applied for 20 min. Afterwards, the slides were incubated for 60 min with the primary anti-ER antibody (clone D07, DAKO), anti-PR antibody (PR 636, Dako at 1:50 dilution); Polyclonal HER2 antibody in the Herceptin kit (HercepTest, DAKO); Ki67 antibody (clone MIB-1, 1:50 dilution; Dako); and Anti-Androgen receptor antibody [EPR1535 (2) (ab133273). The reaction was visualized by incubating the sections with diaminobenzidine (DAB) for 15 min after which Mayer’s hematoxylin was used

### Interpretation of Immunohistochemical Staining

For ER and PR expression, moderate to strong nuclear staining in ≥ 1% of the tumor cells was considered positive. Her2/neu was considered positive if at least 10% of tumor cells exhibited 3+ cell membrane staining. The cut-off point for Ki67 expression was 14%. AR expression was semi-quantitatively scored using an H-score like the method described by Niemeier et al. An immunohistochemical score >10 was considered as a positive result (18).

We analyzed the extended adjuvant treatment after initially proposed protocols of chemotherapy ± radiotherapy, which was reported in patient files and records. Hence, patients were followed in 3 groups: the first received bicalutamide (anti-androgen) in AR positive in 50 mg, with or without meals once daily for 2 years, and group 2 who had negative AR and received capecitabine 650 mg/ m2 BID for one year, and group 3 patients who had unknown AR status and received docetaxel in a protocol of 15mg/ m2 in weekly for 4 weeks to be escalated to 20 mg/m2 once per week with accepted lab consideration for 6 months.

### Statistical Analysis

Continuous variables were expressed as the mean ± SD and median (range), and the categorical variables were expressed as a number (percentage). The percentages of categorical variables were compared using Pearson’s Chi-square test or Fisher’s exact test when appropriate. The trend of change in the distribution of relative frequencies between ordinal data was compared using the Chi-square test for trend. Overall Survival (OS) was calculated as the time from diagnosis to death or the most recent follow-up contact (censored). Disease-Free Survival (DFS) was calculated as the time from the date of surgery to the date of relapse or the most recent follow-up contact when patient was known to be relapse-free. Stratification of OS and DFS was done according to intention to treat (ITT). These time-to-event distributions were estimated using the method of Kaplan-Meier plot and compared using a two-sided exact log-rank test. All tests were two-sided. A p-value <0.05 was considered significant. All statistics were performed using SPSS 22.0 for windows (IBM Inc., Chicago, IL, USA).

## RESULTS

### Clinicopathological Features

The mean age at presentation was 42.60 ±12.05 years. Most of the cases were grade III (56.3%) and 82.5 % showed lymph node metastasis. IDC of no special type was the most common histopathological type (65%). As regards the pathologic stage, 55% of the cases were T1 with lymphovascular invasion in 55% of the cases. The clinicopathological data of the cases enrolled in this study were summarized in (Table 1).

**Table 1:** Clinicopathological features, treatment, and outcome of triple-negative breast cancer patients.

**Characteristics**	**All patients (N=80)**		**Characteristics**	**All patients (N=80)**	
	**n**	**%**		**n**	**%**
**Age (years)**			**Stage**		
Mean ± SD	42.60	±12.05	Stage IB	13	16.3
Median (Range)	41	(22 – 68)	Stage IIA	15	18.8
≤35 years	32	40	Stage IIB	18	22.5
>35 years	48	60	Stage IIIA	10	12.5
**Menopausal status**			Stage IIIB	7	8.8
Premenopausal	45	56.3	Stage IIIC	9	11.3
Postmenopausal	27	33.8	Stage IV	8	10
Perimenopausal	8	10	**Ki-67**		
**Family history**			High	53	66.3
Positive	11	13.8	Low	27	33.8
Negative	69	86.3	**Androgen receptor**		
**Type of surgery**			Positive	21	26.3
MRM	46	57.5	Negative	24	30
BCS	25	31.3	Unknown	35	43.8
No	9	11.3	**CEA**		
**Grade**			High	49	61.3
Grade I	7	8.8	Normal	31	38.8
Grade II	28	35	**CA 15-3**		
Grade III	45	56.3	High	37	46.3
**Pathological subtype**			Normal	43	53.8
IDC	52	65	**Radiotherapy**		
ILC	10	12.5	Yes	71	88.8
Mixed IDC & ILC	15	18.8	No	9	11.3
Others	3	3.8	**Chemotherapy**		
**Extracapsular extension**			AC-Taxanes	54	67.5
Absent	40	50	Carboplatin+Taxanes	13	16.3
Present	33	41.3	EC-Taxanes	13	16.3
Not reported	7	8.8	**Follow-up duration (months)**		
**Lymphovascular invasion**			Mean ± SD	33.80	±4.57
Absent	31	38.8	Median (Range)	36	(16 – 36)
Present	44	55	**Mortality**	(N=72)	
Not reported	5	6.3	Alive	60	83.3
**Lymph node status**			Died	12	16.7
N0	14	17.5	**Recurrence**	(N=72)	
N1mi	13	16.3	Absent	50	69.4
N1	26	32.5	Present	22	30.6
N2	6	7.5			
N3	21	26.3			
**Tumor stage**					
T0	4	5			
T1	44	55			
T2	13	16.3			
T3	16	20			
T4	3	3.8			

**IDC:** Invasive ductal carcinoma, **ILC:** Invasive lobular carcinoma, **MRM:** Modified radical mastectomy, **BCS:** Breast conserving therapy Categorical variables were expressed as number (percentage); Continuous variables were expressed as mean ± SD & median (range).

### The Relation Between Clinicopathological Features and Androgen Receptor IHC Staining

**Figure 1:**
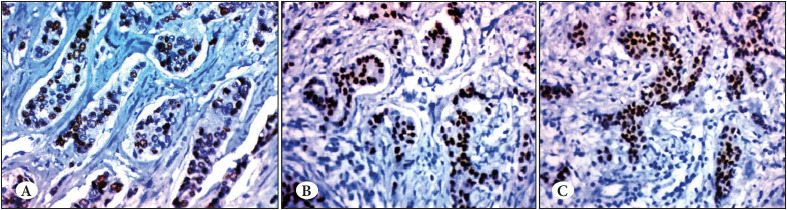
**A)** A case of TNBC grade 1 showing positive androgen expression (IHC; x400). **B)** A case of TNBC grade 2 showing positive androgen expression (IHC; x400). **C)** A case of TNBC grade 3 showing positive androgen expression (IHC; x400).

Positive androgen expression was noted in 26.3% of the studied cases (Figure 1). Negative Androgen expression revealed strong association with younger age ≤35 years, premenopausal status, higher grade, extracapscular extension, lympho-vascular emboli, Ki 67 and CA15-3 with p values (0.003, 0.02, <0.001, 0.001, 0.027, 0.005, 0.009 respectively) (Table 2). Regarding to the toxicity of bicalutamide and capecitabine was shown in (Table 2).

**Table 2:** Relationship between clinicopathological features and androgen receptor IHC staining.

**Characteristics**	**All Patients**		**Androgen Receptor**				**p-value**
	**(N=45)**		**Positive (N=21)**		**Negative (N=24)**		
	**n**	**%**	**n**	**%**	**n**	**%**	
**Age (years)**							
≤35 years	19	42.2	4	21.1	15	78.9	0.003‡
>35 years	26	57.8	17	65.4	9	34.6	
**Menopausal status**							
Premenopausal	26	75	8	30.8	18	69.2	0.028‡
Postmenopausal	17	37.8	11	64.7	6	35.3	
Perimenopausal	2	4.4	2	100	0	0	
**Family history**							
Positive	5	11.1	1	20	4	80	0.352‡
Negative	40	88.9	20	50	20	50	
**Grade**							
Grade I	5	11.1	4	80	1	20	<0.001§
Grade II	19	42.2	14	73.7	5	26.3	
Grade III	21	46.7	3	14.3	18	85.7	
**Pathological subtype**							
IDC	27	60	14	51.9	13	48.1	0.695‡
ILC	6	13.3	3	50	3	50	
Mixed IDC & ILC	10	22.2	3	30	7	70	
Others	2	4.4	1	50	1	50	
**Extracapsular extension**							
Absent	25	55.6	17	68	8	32	0.001‡
Present	20	44.4	4	20	16	80	
**Lymphovascular invasion**							
Absent	16	35.6	11	68.8	5	31.2	0.027‡
Present	29	64.4	10	34.5	19	65.5	
**Lymph node status**							
N0	2	4.4	2	100	0	0	0.928§
N1mi	24	53.3	10	41.7	14	58.3	
N1	4	8.9	1	25	3	75	
N2	12	26.7	7	58.3	5	41.7	
N3	3	6.7	1	33.3	2	66.7	
**Tumor stage**							
T0	10	22.2	4	40	6	60	0.324§
T1	8	17.8	5	62.5	3	37.5	
T2	15	33.3	8	53.3	7	46.7	
T3	6	13.3	4	66.7	2	33.3	
T4	6	13.3	0	0	6	100	
**Stage**							
Stage IB	8	17.8	5	62.5	3	37.5	0.082§
Stage IIA	10	22.2	4	40	6	60	
Stage IIB	12	20.8	7	58.3	5	41.7	
Stage IIIA	6	13.3	4	66.7	2	33.3	
Stage IIIB	5	11.1	1	20	4	80	
Stage IIIC	4	8.9	0	0	4	100	
**Ki-67**							
High	29	64.4	9	31	20	69	0.005‡
Low	16	35.6	12	75	4	25	
**CEA**							
High	27	60	10	37	17	63	0.113‡
Normal	18	40	11	61.1	7	38.9	
**CA 15-3**							
High	20	44.4	5	25	15	75	0.009‡
Normal	25	55.6	16	64	9	36	

**IDC:** Invasive ductal carcinoma, **ILC:** Invasive lobular carcinoma. Categorical variables were expressed as number (percentage); ‡ Chi-square test; § Chi-square test for trend; p<0.05 is significant.

### Toxicity Outcome

Bicalutamide was well tolerated as 17 (81%) patients out of 21 patients had shown no toxicity, only 2 patients showed grade (G) 2 hot flushes, one patient showed weight change in the form of increase in weight, only one patient suffered from G1 drowsness. 14 /27 patients (58.3%) had shown no toxicity of capecitabine proposal, 5(20.8%) patients were presented by G2 diarrhea, 3 patients (12.5%) presented by G1 hand pain, redness and swelling, only 2(8.3%) patients were presented by G2 nausia and vomiting. Regards docetaxel, more toxicity was observed only 7 (25.9%) who had no toxicity. Hematological toxicity was observed in 20 patients (74%), all are G1,2 except 2 patients showed G4 anemia, 8 (29.6%) patients were observed with G 1,2 pleural effusion and 6 (22.2%) patients exhibited G1, 2 hepatotoxicity. All previous manifestations were well controlled by medical treatment and proper observations (Table 3 Table 4).

**Table 3:** Toxicity profile of anti-androgen arm.

**Toxicity profile**	**Anti-androgen arm (N=21)**	
	**n**	**%**
No toxicity	17	81
Hot flashes	2	9.5
Weight changes	1	4.8
Drowsiness	1	4.8

**Table 4:** Toxicity profile of capecitabine arm and docetaxel arm.

**Toxicity profile**	**Capecitabine arm (N=24)**		**Docetaxel arm (N=27)**	
	**n**	**%**	**n**	**%**
No toxicity	14	58.3	7	25.9
Nausea & vomiting	2	8.3	0	0
Hand pain redness & swelling	3	12.5	0	0
Diarrhea	5	20.8	3	11.11
Hematological	2	8.3	20	74
Pleural effusion	0	0	8	29.6
Hepatotoxicity	0	0	6	22.2
Nephrological toxicity	0	0	3	11.11

### Survival Outcome

**Figure 2:**
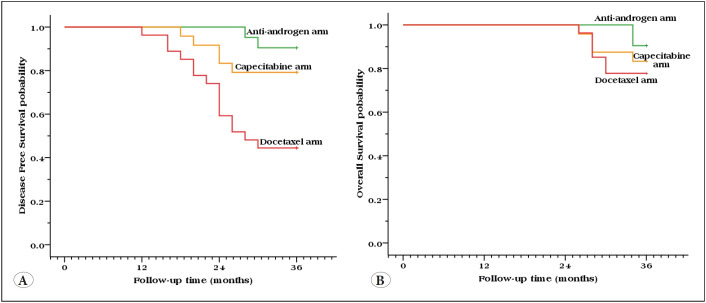
Kaplan Meier plot of the studied arms, Left panel: Disease-free survival **(A)**, Right panel: Overall survival **(B)**.

The mean 3 years DFS was 35.3 months in patients who received bicalutamide, 33.16 months in patients received capecitabine, while in the docetaxel arm was 28.2 months with significance *P*=0.001, better DFS was in the favor of bicalutamide administration. 3 years overall survival (OS) in patients who received bicalutamide better than those received capecitabine or docetaxcel but of no significance *P*=0.46 (Table 5 and Figure 2).

**Table 5:** Comparison between anti-androgen arm, capecitabine arm, and docetaxel arm regarding survival outcome.

**Outcome**	**All patients (N=72)**			**Anti-androgen Arm (N=21)**			**Capecitabine Arm (N=24)**			**Docetaxel** **Arm (N=27)**		**p^1^**	**p^2^**	**p^3^**	**p^4^**
	**n**	**%**		**n**	**%**		**n**	**%**		**n**	**%**				
**Recurrence**															
Absent	50	69.4		19	90.5		19	79.2		12	44.4	0.001‡	0.422‡	0.001‡	0.011‡
Present	22	30.6		2	9.5		5	20.8		15	55.6				
**DFS**															
Mean DFS (95%CI)	31.94 months (30.41-33.47)			35.33 months (34.44-36.22)			33.16 months (30.89-35.44)			28.22 months (25.27-31.17)		0.001†	0.264†	0.001†	0.012†
12-month DFS	98.6%			100%			100%			96.3%					
24-month DFS	79.2%			100%			83.3%			59.3%					
36-month DFS	69.4%			90.5%			79.2%			44.4%					
**Mortality**															
Alive	60	83.3		19	90.5		20	83.3		21	77.8	0.504‡	0.670‡	0.437‡	0.731‡
Died	12	16.7		2	9.5		4	16.7		6	22.2				
**OS**															
Mean OS (95%CI)	34.91 months (34.29-35.53)			35.81 months (35.55-36.06)			34.83 months (33.68-35.98)			34.29 months (33.06-35.52)		0.468†	0.444†	0.212†	0.629†
12-month OS	100%			100%			100%			100%					
24-month OS	100%			100%			100%			100%					
36-month OS	83.3%			90.5%			83.3%			77.8%					

**DFS:** Disease free survival; **OS:** Overall survival Continuous variables were expressed as mean (95%CI); categorical variables were expressed as number (percentage); ‡ Chi-square test; † Logrank test; **p^1^:** p-value for the test between the three arms; **p^2^:** p-value for the test between the anti-androgen arm and capecitabine arm; **p^3^:** p-value for the test between anti-androgen and docetaxel arm; **p^4^**: p-value for the test between capecitabine and docetaxel arm; p<0.05 is significant.

## DISCUSSION

In the current study, we investigated the clinic-pathological criteria of triple-negative breast cancer patients who were conducted at our institutes at a specific period with high lighting on the proposed treatment such as antiandrogen (bicalutamide), capecitabine and docetaxel as an extended treatment

In this study we found that negative androgen expression had shown strong associations with younger age (≤35 years), premenopausal status, higher grade, extracapsular extension, lymphovascular invasion, Ki 67, and CA15-3, this was agreed with Farag et al. who studied the prevalence of androgen receptor expression in 90 patients of TNBC and the criteria of their clinic-pathology with no treatment proposal and revealed that AR negative patients was significantly associated with higher grade, higher stage, lymph node metastasis, distant metastasis, vascular, perineural invasion and high CA15-3 (19).

In our study 71/80, patients received adjuvant radiotherapy post adjuvant chemotherapy protocols such as AC-Taxens, carboplatin-Taxens, and EC-Taxens. Pal et al. confirmed the efficacy of combination treatment for TNBC patients (20). These findings were focused on evidence-based treatment recommendations (12,21,22). Furthermore, Chen et al. reported that radiotherapy post-mastectomy was associated with more prognosis improvement (23).

We agreed with Zakaria et al. who reported in their study with inclusion of 77 TNBC patient that the median age was 35.6 with a range of (19-63) and 21∕ 77 patients (27.2%) were AR positive. AR expression was associated with high grade, high KI 67, positive nodal status and CA15-3. Along with her study, nobody died in AR positive patients, these patients received bicalutamide 50 mg once daily over 2 years as treatment duration with better 2 and 3 year OS which were 85% and 78% with p values of <0.001, 0.0005 respectively; bicalutamide was well-tolerated toxicity, no grade 3 and 4 adverse events in TNBC AR positive patients as well as 6 (28.57%) out of 21 patients presented in the form of 3 patients presented with nausea, two patients presented with breast fullness, tenderness, and hot flushes and only one patient who was presented by weight gain but with better OS and DFS outcome (11). In our study we found that bicalutamide was well tolerated as 17 (81%) patients out of 21 patients had shown no toxicity, only 2 patients showed G2 hot flushes, one patient showed weight change in the form of an increase in weight, and only one patient suffered from G1 drowsiness. These were all tolerated with more affordability. The mean 3-year DFS was 35.3 months in patients who received bicalutamide as extended treatment and better OS.

In our study, 13/27 patients (42.7%) had shown toxicity to capecitabine proposal, 5 (20.8%) patients presented with G2 diarrhea, 3 patients (12.5%) presented with G1 hand pain, redness and swelling, and only 2 (8.3%) patients presented with G2 nausea and vomiting while hematological toxicity was observed in 2 (8.3%) patients. In the docetaxel arm, unfortunately 20 (74%) patients exhibited hematological toxicity, and 8 (29.6%) patients complained of pleural effusion G1,2 where the pattern of toxicity was milder than reported by Abdelaziz et al. investigated 22 patients with TNBC who received metronomic capecitabine as extended treatment in non-metastatic condition, common toxicities were in the form of 2 patients presented by G1/ G2 hand foot syndrome and another 2 patients presented by G3/4 hand foot syndrome, 3 patients presented by G1/2 nausea and vomiting and 2 patients presented by G3 diarrhea, on the other hand hematological toxicity was observed in 5 patients in the form of anemia, in our study The 3 years DFS was 79.2%, 3 years OS 83.3% that was near to what was reported by Abdelaziz et al. as 3 years OS was 86.4%, while 3 year DFS was 81%.22; (24) in contrary to Alagizy et al., who studied 41 patients of TNBC and reported higher adverse effects of extended metronomic capecitabine after adjuvant chemotherapy such as G1 palmar– plantar erythrodysesthesia in 13 patients (31.7%); G 1 diarrhea in five patients (12.2%); and G1 vomiting in two patients (4.9%) with no G3∕ 4 adverse effects. follow-up mean disease-free survival (DFS) was 42.4 months (95% CI). overall survival was 44.34 months (95% CI) with a lower incidence of recurrence and distant metastasis in comparison to other studies (25).

Abdelmaksoud et al. investigated the role of docetaxel in an extended treatment of 31 patients with triple negative breast cancer with a 3-year DFS and OS of 56.4 and 78.1, respectively, and a tolerable toxicity profile, and encouraged the use role of metronomic docetaxel for better survival gains (16).

TNBC patients may benefit from antiandrogen treatment as it is well tolerated with significantly lower toxicity than that of chemotherapy, and it can be proposed with other agent combinations (8,26–28). We agree with Locatelli et al. who stated that low dose maintenance capecitabine was an attractive approach with low cost, good tolerability, and manageability, especially in high-risk disease (29).

Inhibitors of such pathways as CDK4/6, PI3K, RAS, and MEK which command cell cycle progression, survival, proliferation, invasiveness, and drug escape can be optimally combined with an AR antagonist (30–32).

Our recommendation is to encourage further collaborative studies on a larger number of studied cases to gain more accurate information in the absence of data bias with searching for novel, easy and cheap methods and aiming for proper treatment strategies and a proper patient selection guidance philosophy, targeting each triple-negative phenotype in different clinical scenarios.

In conclusion, progress in the treatment of TNBC remains an important challenge. The proposed bicalutamide shows better outcomes in favor of OS and toxicity with better tolerability. On the other hand, metronomic capecitabine is well tolerated with accepted patient compliance and affordability compared to docetaxel and is warranted for problem solving with better disease-free survival and overall survival in some triple-negative breast cancer patients.

## Conflict of INTEREST

The authors declared that there is no conflict of interest.
